# A Text-Book of Human Physiology

**Published:** 1888-10

**Authors:** 


**A Text-Book of Human Physiology.** By AUSTIN FLINT, M. D., LL. D., Professor of Physiology and Physiological Anatomy in the Bellevue Hospital Medical College, New York; Visiting Physician to Bellevue Hospital, etc., etc. With 316 figures in the text, and two plates. Fourth edition; entirely rewritten. Large 8vo; pp. xvii; 872. New York: D. Appleton & Co. 1888.

Dr. Flint has long been recognized as a teacher of distinction upon the subject of physiology. A new work, which this practically is, from his pen is of importance to the student of medicine. The author is frank enough to say that on account of the advancement made in this department he has been unable to follow closely in teaching the edition of 1880, the defects of which were so important that they could only be remedied by making a new book. This shows a most commendable desire to have his text-book equal the best and to be abreast of the times. We are inclined to think that he has accomplished both these purposes. The mention of a few of the changes in this edition will indicate this. The historical references have been curtailed, as they should be in a book designed as a text-book; the new chemical nomenclature has been adopted; the advances in minute anatomy as bearing upon physiology have been incorporated; elaborate description of apparatus and methods has been avoided, as belonging rather to the laboratory than to a work of this kind; the equivalent of the English weights and measures have been given in the metric system; many illustrations have been left out and others substituted, and the defects in all of them carefully corrected. Besides this, the form and typography of the book have been changed and very much improved. The type is larger and clearer, and consequently much less tiresome. We remember many of the difficulties we experienced in the use of the first edition, and we feel confident that they will not occur with this one. We cannot close this short notice without saying something of the work of the publishers They have done their part so well, they have made such a beautiful book, that too much praise cannot be said of it. Any author should feel proud to have his work thus presented to the public.

